# COVID‐19 induced anorexia nervosa: A case series and brief review of literature

**DOI:** 10.1002/ccr3.7534

**Published:** 2023-06-13

**Authors:** Soode Tajik Esmaeeli, Zahra Rahimi, Fahimeh Saeed, Sheikh Shoib

**Affiliations:** ^1^ Department of Psychiatry, Minimally Invasive Surgery Research Center, Rasool‐e Akram Hospital, Mental Health Research Center, Psychosocial Health Research Institute, School of Medicine Iran University of Medical Sciences Tehran Iran; ^2^ School of Medicine Iran University of Medical Tehran Iran; ^3^ Psychosis Research Center University of Social Welfare and Rehabilitation Sciences Tehran Iran; ^4^ Department of Health Services Jawahar Lal Nehru Memorial Hospital Srinagar Kashmir India

**Keywords:** anorexia nervosa, COVID‐19, eating disorder, mental health, SARS‐CoV‐2

## Abstract

**Key Clinical Message:**

This article emphasizes that patients presenting with COVID‐19 infection especially presenting with predominantly gastrointestinal symptoms and a history of eating disorder or even other mental disorders should be evaluated thoroughly and differential diagnoses should be considered. Clinicians should keep in mind that eating disorders may happen after COVID infection or vaccination.

**Abstract:**

The emergence and global spread of the 2019 novel coronavirus (COVID‐19) have caused a significant mental health burden on communities around the world. Factors related to COVID‐19 affect mental health in the general community, but may have more adverse effects on individuals with pre‐existing mental illnesses. Additionally with the new living conditions and increased focus on hand hygiene and fear of contracting COVID‐19, depression, anxiety, and obsessive–compulsive disorder (OCD) are more likely to be exacerbated. Eating disorders such as anorexia nervosa (AN) have exhibited an alarming increase due to social pressure especially through social media. Also, many patients reported relapses since the commencement of the COVID‐19 pandemic. We report five cases of AN that developed or exacerbated after COVID‐19 infection. Four patients have newly developed (AN) and one case relapsed after COVID‐19 infection. Also, one of the patient's symptoms exacerbated after remission following a COVID‐19 vaccine shot. The patients were managed medical and non‐medically. Three of cases have reported improvements while two other cases were lost because of poor compliance. It might be possible that people with history of eating disorder, or other mental disorders may be more susceptible to newly develop or exacerbate eating disorders after COVID‐19 infection especially when symptoms are gastrointestinal dominant. There is currently minimal evidence on the specific risk of COVID‐19 infection in patients with AN and reporting cases of AN after COVID‐19 infection could help learn the risk, prevent and manage patients. Clinicians should keep in mind that eating disorders may happen after COVID infection or vaccination.

## 1 INTRODUCTION

The emergence and global spread of the 2019 novel coronavirus (COVID‐19) have caused a significant mental health burden on communities around the world.^1^ Many of the psychiatric manifestations of COVID‐19 are the result of psychological stressors, such as fear of illness and death, prolonged social isolation, and uncertainty and fear about the future.^1^ However, there is increasing evidence suggesting that the virus itself can cause neuropsychiatric symptoms among infected individuals including encephalopathy, anxiety, depression, mania, and trauma‐related disorders.^1^

Anorexia nervosa (AN) is an eating disorder characterized by restrictive eating and an intense fear of weight gain and is associated with psychiatric disorders, secondary amenorrhea, nutritional damage, impaired endocrine panel as well as cardiac, gastrointestinal, and hematological complications.^2^, ^3^ Eating disorders such as AN have shown an alarming increasing incidence due to social pressure especially through social media.^4^, ^5^, ^6^ This increasing incidence has been reported for the last few decades but especially during the last 6 months of 2020 when it has become a challenging issue due to the pandemic period. Lockdown recommendations refocused the population on viral content which aggressively forced unrealistic models of weight and body image.^7^, ^8^ It is reported that there has been a 104% increase in children with AN requiring admission to the hospital for nutritional rehabilitation compared with the three previous years.^2^ There is currently minimal evidence on the specific risk of COVID‐19 infection in AN patients. Patients who present with COVID‐19 infection, and have a history of eating disorders or other mental disorders, should be thoroughly evaluated and differential diagnoses should be considered. We are reporting five cases with a history of COVID‐19 infection who developed AN or their symptoms exacerbated.

## 2 CASE PRESENTATION

Five cases were admitted to Rasoul Akram hospital with AN symptoms with positive history of COVID‐19 infection. Patients' histories and data are summarized in Table 3.

### 2.1 Case 1

A 43‐year‐old woman from Miyaneh, resident of Tehran, married, mother of two children, with a bachelor's degree, housewife, was admitted to Rasoul Akram hospital with nausea and vomiting and was severely cachectic. She stated that symptoms started 20 months ago, 10 days after a mild SARS‐CoV‐2 infection with symptoms such as anorexia, nausea, vomiting, and diarrhea. Even after recovery (10 days) from the SARS‐CoV‐2 and complete relief of symptoms, the symptoms of anorexia, nausea, and vomiting remained and become so severe that in recent months she has vomited even after drinking water and fluids. The patient vomits more than 15 times a day and, does not tolerate oral solids and fluids. She has lost 13 kg and reached a weight of 31 kg with a height of 168 (body mass index (BMI) = 10.9) despite the fact that all diagnostic tests and workups were normal in terms of the etiology of vomiting. She has been admitted due to heart problems (Ejection fraction <40%), severe coagulation disorders, electrolyte disorders, severe decrease in serum albumin (below 1), liver cirrhosis, and amenorrhea during last year. On examination, the appearance of the body is cachectic and has bilateral temporal atrophy and no suitable muscle mass for injection. Psychomotor slowing is observed. The patient's attitude was semi cooperative. Mood of the patient was euthymic and had a restricted affect. No hallucinations were found. The form of thought is normal. In the content of her thoughts, she has delusional beliefs about her daughter and has obsessive thoughts. Attention, concentration, and cognition are normal. She has poor insight. No recent stressors were obtained during psychiatric interviews that may have contributed to the exacerbation of her AN symptoms. In her previous psychiatric history, she has a history of obsessive–compulsive disorder (OCD) since the age of 18 (washing, symmetry, order, and checking). She also has obsessive–compulsive personality traits. She has a history of mental engagement with weight and body appearance since adolescence, has never been overweight, and was always careful in terms of calorie intake. She also had a history of depression and AN 5 years ago for a year, following her second pregnancy, when the first fetal screening suggested that the fetus may be defective. She had experienced symptoms including severe food restrictions, vomiting, and diarrhea and was ordered to receive shock therapy and psychiatric hospitalization, but the patient refused to be hospitalized or undergo any therapy until after a year that symptoms gradually resolved spontaneously. She has no history of drug use or suicide. The patient has a family history of obsession in her mother and sister. Her husband is very supportive and has a good interpersonal relationship with his wife.

The patient never agreed to treatment, and her husband and family, despite all the recommendations and warnings by the psychiatrist and attending physician, did not agree to the psychiatric hospitalization due to the patient's lack of consent. The patient was prescribed olanzapine 5 mg orally, each night, by psychiatry service during her two‐week hospitalization in the heart department, but due to frequent vomiting, it was difficult to administer the drug orally. In addition, due to severe cachexia, it was not possible to administer the drug intramuscularly. Due to heart problems, the cardiology department did not allow the administration of intravenous haloperidol. On the contrary, the nutrition consultant could hardly and with high risk start TPN feeding for the patient due to advanced liver cirrhosis. The patient was put on the liver transplant list, and the coagulation and electrolyte disorders were partially corrected. The patient was considered to have a very poor prognosis in terms of all departments, including cardiology, gastroenterology, internal medicine, and nutrition. After being discharged from the hospital, the patient did not continue her psychiatric treatment and did not return to the hospital.

### 2.2 Case 2

A 16‐year‐old female student, single, born, and living in Kurdistan, has been admitted to the gastroenterology ward with complaints of repeated vomiting and has been asked for Liaisons' consultation in diagnosing AN. Symptoms started in May the year before, 1 week after a moderate SARS‐CoV‐2 infection and were mainly nausea, vomiting, and diarrhea. The primary COVID‐19 symptoms resolved after 2 weeks. At that time, she intentionally induced vomiting for 2 months to get rid of nausea, but after that and until now, vomiting occurs spontaneously. During the last 6 months, she lost 15 kg of weight and now weighs 55 kg and her height is 161 cm (BMI = 21.1). During this time, she has had three periods of amenorrhea in a row and her menstrual periods are irregular. She mentioned that she has always had a lot of mental engagement with her weight, but has never been able to diet and lose weight until the recent COVID‐19 infection. She states that she is currently satisfied with her weight loss and vomiting and mentions that even if the vomiting is treated, she will not eat anything to stay slim. She also mentioned recent emotional stress (a year ago) which was the severance of a relationship with her only best friend. She had been overweight since childhood and has always been ridiculed by her classmates at school. According to her, she has always been a diligent student and has obtained the highest scores to compensate for the ridicule caused by her weight, but she is still disliked by her peers. She has had a history of binge‐eating attacks since he was a child. She also has had major depression disorder (MDD) during school.

She has a younger sister who is well‐proportioned. She does not have a close relationship with her parents. She has controlling and high expressed emotion parents. Her parents are also in a normal weight range and constantly used to blame her for gaining weight and overeating. She is obsessed with schoolwork and spends a lot of time on even the simplest tasks. Obesity and obsession are common in the paternal family. She has no mood symptoms at the moment.

The patient had an appropriate state of selfcare. Psychomotor and speech were normal. Her attitude was cooperative. She had a euthymic mood and appropriate affect. Perception and thought form were normal. Obsessive thoughts were noted in content of thought. Attention, concentration, and cognitive status were good. She has relatively poor insight.

There were no abnormal medical findings in the diagnostic examinations, including endoscopy. Echocardiogram, endoscopy electroencephalogram (EEG), and brain electroencephalogram (MRI) were normal. Mirtazapine was prescribed for the patient, and psychotherapy was started. During the 7 days of hospitalization, the symptoms of nausea and vomiting decreased to some extent, and she was discharged with a good general condition. The patient continued psychiatric treatment in her city and was also treated for obsession. Family therapy was also recommended for the patient.

### 2.3 Case 3

A 15‐year‐old male student from Sirjan had been admitted to the gastrointestinal ward with frequent complaints of nausea and vomiting. For the past 7 months, following a moderate COVID‐19 infection with symptoms of anorexia and nausea, he has suffered from recurrent vomiting, which recently occurs spontaneously, even with fluids. He made a recovery from the COVID‐19 infection after 12 days. During the last 7 months, he lost about 11 kg. Currently, his weight and height are 57 and 159, respectively (BMI = 22). He had a dysphoric mood with no thoughts of death or suicide. He was interested in snacks and fast food and high‐calorie foods such as chips and pizza. Addiction history was negative. He was always overweight before getting infected with Covid and was ridiculed at school by his peers. Has no history of Bulimia or anorexia. He has a history of obsessive–compulsive disorder (contamination, order, and symmetry), which has become more severe after COVID‐19. He has suffered from a decline in school performance in recent months. No apparent conflict was found in the family environment and interpersonal family relationships. No psychiatric history was found in the family. All diagnostic tests, including endoscopy, were normal. His laboratory data showed electrolyte disturbance (hypokalemia K = 2.8, Mg = 1.8).

After pharmacological and non‐pharmacological treatment and hospitalization for 10 days, the patient's symptoms were under control for the next 6 months, but his AN symptoms relapsed again after the injection of the corona vaccine (Sinopharm) in a milder form. Before infection with covid, the severity of obsessive symptoms was moderate. After being infected with covid, the intensity of obsessive symptoms has become severe. Before and after the injection of the vaccine, the intensity of obsessive symptoms was moderate. Currently, he is still under psychiatric treatment. Obsessions and mood are better, academic function is improved, and symptoms are under control.

### 2.4 Case 4

A previously healthy 13‐year‐old male student from Bandar Abbas has been suffering from recurrent nausea and vomiting for 9 months after a COVID‐19 infection causing symptoms like anorexia, nausea, vomiting, body aches, which has resulted in weight loss of about 14 kg, and weakness and lethargy. The symptoms started 5 days after a severe COVID‐19 infection. He did not required hospitalization for the COVID‐19 symptoms and was recovered after 2 weeks. According to him and his mother, he has been consuming “only water” for the last 2 months and cannot bear food. He currently weighs 54 kg and is 157 tall (BMI = 21). Since childhood, he has had a constant mental occupation with his weight due to being overweight and has always been ridiculed and harassed at school. He has a history of binge eating but no bulimia or anorexia. He has never been able to follow a diet and lose weight, and he is happy with the anorexia caused by Covid. He also has a history of facial dysmorphia. He has two older brothers with whom he has good relationships. His parents have a good relationship with the patient, and the family atmosphere is good. He is the only family member who was ever overweight. Due to the low average weight of the people of the southern region of the country, his slight overweight (BMI = 27) has been very significant in the eyes of others.

Due to serum therapy, he had no symptoms of dehydration at the time of examination. Endoscopy and colonoscopy were reported to be normal. In the patient's tests, there were no other findings except the findings related to anorexia.

Drug treatment with Mirtazapine 7.5 and psychotherapy were started for the patient, and after 10 days and completing the evaluations, he was discharged with a good general condition, and the severity and frequency of vomiting decreased. The patient was referred to a psychiatrist in his city, and the family was educated on the necessity of follow‐up treatment.

### 2.5 Case 5

Thirty‐four‐year‐old man, single, postgraduate and currently unemployed, is admitted to the nephrology department due to severe electrolyte disturbances, severe anemia (Hb: 6, RBC: 2), and malnutrition. The patient had been having gastrointestinal symptoms 3 days after a moderate COVID‐19 infection 2.5 years ago, which has resulted in 35 kg of weight loss during this period. The COVID‐19 symptoms were resolved in 10 days but he continued to experience nausea, vomiting, and weight loss. He currently weighs 44 kg with a height of 178 (BMI = 13.8). He mentions that he feels bloated and his stomach cannot digest food. Because of the fear of indigestion, he does not eat any food during the day, but at night he consumes a large amount of food impulsively and immediately bends forward and vomits all the food he has eaten. It should be noted that until a few months ago, he induced vomiting by stimulating the gag reflex, but recently it occurs without induction. He complains of this disproportionate weight loss, but believes the reason is the inability of his stomach to digest food, which occurred after the COVID‐19 infection. In previous records, there has never been a history of weight gain or weight loss. He has a history of bulimia nervosa 10 years ago and has always been preoccupied with the issue of body weight and appearance. 5 years ago, he went on a heavy diet, exercised heavily, did not eat during the day, and ate a lot of food at night, but did not lose weight (82 kg). He has a history of illness anxiety and has always been worried about getting a serious illness with getting the slightest physical symptoms. During his COVID‐19 infection, despite having only gastrointestinal symptoms and no lung involvement, he had a strong fear of death. He still has a lot of mental occupation with stomach cancer. He has a history of OCD since adolescence (washing and order). He got married at the age of 25 and divorced 3 years later, and now, he feels very guilty about harassing his ex‐wife. He is currently unemployed. He lives with his parents, and the expenses are covered by them. His father is an aggressive and nervous person and has always had a limited relationship with him. The mother is a controlling, blaming individual with high expressed emotion. Emotional support in the family is weak.

Since 1 year ago, he has had a history of two hospitalizations in the internal department due to electrolyte disorders and malnutrition where he did not continue drug treatments and has had poor treatment compliance.

In examination, he looks cachectic and has a depressed mood. None of the endoscopy and colonoscopy findings showed abnormalities justifying his symptoms.

Oral olanzapine and psychotherapy were started for the patient during hospitalization. Due to the severe cachexia of the patient, it was not possible to administer the drug by injection. During nutrition counseling, he was given intravenous TPN. After 18 days and partial correction of electrolytes, he was discharged. After discharge, he underwent psychoanalytic psychotherapy, family therapy, and obsession therapy and the symptoms were partially improved, but after 20 sessions of psychotherapy, despite pursuit by the therapist, the patient abandoned the treatment.

## 3 DISCUSSION

Anorexia nervosa (AN) is an eating disorder characterized by restrictive eating and a severe fear of gaining weight. It is a complex condition which includes psychiatric disorders, secondary amenorrhea, nutritional damage, impaired endocrine panel up to bone loss as well as cardiac, gastrointestinal, and hematological complications.^9^ Most complications can be resolved with early effective treatment.^10^ Treatment generally involves restoration of weight and proper nutrition which leads to normalization of blood counts over time, altering the caloric sources to a softer more liquid consistency, smaller more frequent meals, and ongoing weight gain to alleviate gastric symptoms and help hepatic apoptosis.^3^ Any organ can actually be affected by the disease; therefore, there is a need for a multidisciplinary team in addition to multiple hospitalization episodes.^9^ Not only long‐term requirements but also a high mortality rate have been described in relation to malnutrition and weight loss.^9^, ^10^

Definite diagnosis of AN is made by either DSM‐5 criteria (Table 1) or ICD‐10 (Table 2) diagnostic criteria for AN. Based on both of these criteria, all five cases are confirmed AN cases.

**TABLE 1:** DSM5 criteria for anorexia nervosa.

A	Limiting of calorie intake relative to requirements, leading to significantly low body weight for the patient's age, sex, developmental trajectory, and physical health. Significantly low weight is defined as a weight that is less than the minimal normal weight (e.g., BMI ≤17.5) or, in children and adolescents, less than the minimal expected weight
B	Severe fear of gaining weight or of becoming fat, or persistent behavior that interferes with weight gain, even if the patient's weight is significantly low
C	Disturbance in the way in which one's body weight or shape is experienced, undue influence of body weight or shape on self‐evaluation, or persistent lack of recognition of the seriousness of the current low body weight.^11^ *These three criteria are all required for a diagnosis of anorexia nervosa^11^

**TABLE 2:** ICD‐10 criteria.

A	Weight loss, or in children a lack of weight gain, leading to a bodyweight of at least 15% below the normal or expected weight for age and height
B	The weight loss is self‐induced by avoidance of “fattening foods”.
C	A self‐perception of being too fat, with an intrusive dread of fatness, which leads to a self‐imposed low weight threshold.
D	A widespread endocrine disorder involving the hypothalamic–pituitary‐gonadal axis, manifest in the female as amenorrhea, and in the male as a loss of sexual interest and potency (an apparent exception is the persistence of vaginal bleeds in anorexic women who are on replacement hormonal therapy, most commonly taken as a contraceptive pill).
E	Does not meet criteria A and B of bulimia nervosa^11^

**TABLE 3:** Patients' histories and data.

	Case 1	Case 2	Case 3	Case 4	Case 5
Age (years)	43	16	15	13	34
Sex	Female	Female	Male	Male	Male
Bodyweight (kg)	31	55	57	54	44
Body mass index (kg/m^2^)	10.9	21.2	22	21	13.8
Disease duration	20 months	19 months	7 months	9 months	2.5 years
Weight loss (kg)	13	15	11	14	35
Time between COVID & AN symptoms	10	7	Simultaneously	5	3
COVID‐19 severity	Mild	Moderate	Moderate	Severe	Moderate
COVID recovery time	10	14	12	14	10
Hospitalization time for AN	14	7	10	10	18
Past psychiatry history	Psychosis, Depression, anorexia nervosa, OCD	Binge eating, bulimia nervosa, MDD	Binge eating, OCD	Binge eating, BDD	Illness anxiety, OCD, bulimia nervosa, MDD
Comorbid psychiatric disease	OCD, OCPD traits	OCD	OCD	BDD	Illness anxiety, OCD
Family history	OCD in sister and mother	Obesity/overweight and OCD in paternal family	–	–	High expressed emotions, overcontrolling parents, OCD and OCPD in mother
Amenorrhea	Yes	Yes	—	—	—
K (mmol/L)	2.5	3.2	2.8	4.3	2
AST (U/L)	151	37	25.3	29	58
ALT (U/L)	162	10	33.6	29.7	67
Alkaline P (IU/L)	958	226	204	176	N/A
Mg (mg/dL)	1.1	2.8	1.8	1.9	2.2
Ca (mg/dL)	7.6	10.2	9.3	9.5	7.3
Lipase (U/L)	N/A	34	28	22	N/A
Amylase (U/L)	N/A	58	52	37	N/A
BUN (mmol/L)	N/A	7	5.5	6.2	N/A
Creatinine (μmol/L)	N/A	1.1	0.8	0.9	1.4
Hb (g/dL)	6.4	13	12.1	12.7	6
RBC (million/mm^3^)	3.1	4.2	5.2	4.8	2
Albumin (g/dL)	<1	5.1	4.7	3.9	<1.5
TSH (mIU/L)	16.2	3.1	2.5	2.7	22
ESR (mm/h)	80	4	9	7	82
CRP (mg/dL)	>24	N/A	N/A	N/A	N/A
PT (s)	27.3	9.7	8.9	11.2	25.8
PTT (s)	42.1	26	31	28	2.3
INR	2.52	0.9	0.9	0.8	46.7
Toxicology	Negative	Negative	Negative	Negative	Negative
Medical comorbidities	Liver cirrhosis, heart failure, hypothyroidism	None	None	None	Low zinc and copper, hypothyroidism

Abbreviations: BDD, body dysmorphic disorder; MDD, major depression disorder; OCD, obsessive–compulsive disorder; OCPD, Obsessive–compulsive personality disorder.

Also, in DSM‐5, severity of AN is classified along four levels by use of the individual's BMI: extreme (BMI <15 kg/m^2^), severe (BMI 15–15.99 kg/m^2^), moderate (BMI 16–16.99 kg/m^2^), and mild (BMI ≥17 kg/m^2^).^11^

Furthermore, the Eating Disorder Diagnostic Scale^12^ is a 22‐item self‐report questionnaire designed to measure AN, Bulimia nervosa, and Binge‐eating disorder symptomatology aligned with the DSM‐IV diagnostic criteria. Based on DSM‐IV, height and weight data on EDDS Items 19 and 20 that result in (A) A body mass index (BMI = Kg/m^2^) of less than 17.5, (B) A fear of weight gain or becoming fat as indexed by a score of four or greater on EDDE Item 2, (C) Undue influence of body weight or shape on self‐evaluation as indexed by a score of 4 or greater on either EDDS Item 3 or 4, and (D) Amenorrhea in post‐menarcheal females as indexed by a 3 on EDDS Item 21.^12^ Patient 2, 3, and 4 BMI was greater than 17.5 so did not fully meet the EDDS criteria. Patients 1 and 5 fully satisfied the criteria.

Eating disorders such as AN have exhibited an alarming increase due to social pressure especially through social media.^9^ This increase seems to be accelerated during the pandemic.^7^, ^8^, ^9^, ^13^

Both the infected and non‐infected population might be prone to specific experiences, as a result of widespread anxiety, social isolation, stress in healthcare workers and other essential workers, unemployment and financial problems.^1^ Other experiences may be specific to infected individuals, such as concern about the outcome of their illness, stigma, and amnesia or traumatic memories of severe illness.^1^ Also, neuropsychiatric consequences—that is, mental disorders that are the sequelae of brain damage or disease—might be due to the direct effects of viral infection (including brain infection), cerebrovascular disease (including in the context of a procoagulant state), the degree of physiological compromise (e.g., hypoxia), the immune response, and medical interventions.^1^

People with eating disorders may face unique risks secondary to the pandemic. Anxiety about food availability for those with AN or avoidant/restrictive food intake disorder, changes in the access to treatment and social support, isolation, and economic factors that contribute to treatment progress, can lead to possible relapse.^7^

Psychiatric components of AN involving body image anomalies and harmful behaviors that promote weight loss (weight loss of 15% or more or BMI less than 17.5 kg/m^2^) are associated with secondary amenorrhea which underlines central hypogonadism.^14^ The loss of adipose tissue due to a hypercatabolic status acts as an endocrine disruptor and impairs communication with the hypothalamus–pituitary–thyroid, adrenal, and ovarian axes.^14^ For example, achieving menstruation in a young adult female requires at least 20.5% body fat mass.^15^ These pathways are also destroyed by stress, an epigenetic factor in AN (e.g., the new pandemic).^14^ Both of our female patients (cases 1 and 2) experienced amenorrhea even though one of them had a BMI greater than 17.5 kg/m^2^ (BMI in case 2 is 21.2 kg/m^2^).

Factors related to COVID‐19 affect mental health in the general community, but may have more adverse effects on individuals with pre‐existing mental illnesses.^7^ Two of our patients (cases 2 and 4) had histories of binge‐eating episodes. Two patients (numbers 2 and 5) had reported histories of bulimia nervosa. Additionally, four cases (numbers 1, 2, 3, and 5) were diagnosed with OCD. Other pre‐ or co‐existing conditions were psychosis, depression, and illness anxiety. Studies have shown that AN and bulimia nervosa are both associated with many psychiatric comorbidities, mostly Axis I disorders, such as depression, OCD, substance abuse, and personality disorder.^16^, ^17^ Additionally with the new living conditions and increased focus on hand hygiene and fear of contracting COVID‐19, depression, anxiety, and OCD are more likely to be exacerbated.

Termorshuizen et al. stated that patients with pre‐existing eating disorders may experience worsening of symptoms and those with eating disorders in the past may be at risk for recurrence during COVID‐19.^7^ While individuals with bulimia nervosa had exacerbated binge‐eating behaviors, anorexic patients were associated with an increased compensatory physical exercise, and patients with both conditions had more frequent episodes of recurrence during the pandemic outbreak if the condition was previously remitted.^13^ In our report, two of our patients (case numbers 1 and 3) experienced recurrence after remission. Case number 1 was symptom free for 4 years before COVID‐19 infection. Symptoms of case number 3 were controlled by medication and therapy until his vaccine shot when symptoms relapsed in a milder form. There have been a few reports on how the pandemic triggered the onset of eating disorders^18^ and how the number of inpatient and outpatient admission is increased during this time.^8^ Also, many patients reported relapses since the commencement of the COVID‐19 pandemic.^7^ The articles mostly explored new living conditions, social distancing, self‐isolation, changes in food access, more intense use of social media platforms, disruption of daily habits, and more difficult access to healthcare practitioners.^9^

Three patients were teenagers (cases 2, 3, and 4) who could be affected by the viral infection, school closure, and more screen time. School closure and quarantine can adversely affect children's physical health and psychological well‐being. When children stay at home and reduce their outdoor activity, they become less physically active and become socially withdrawn from their usual environment and devoid of peer relations. They are more likely to spend much more time viewing screens, have irregular sleep patterns, and have less favorable diets.^19^

Early pandemic data showed that coronavirus disease restrictions (not the disease itself) impaired the access to eating disorder services while changes of routine activities brought supplementary stress to the subjects.^20^ This statement can be now put to question since all our five patients experienced symptoms after COVID‐19 infection.

## 4 CONCLUSION

Anorexia nervosa is a severe condition affecting every organ of the body and should be considered during the COVID‐19 pandemic period because of a higher risk of relapse associated with the new living conditions. There might be a connection between AN and COVID‐19 although it is not clear how. The consequences of pandemic period at a community level or indirect effects of COVID‐19 infection at an individual level or direct effect of virus could partly explain this. It is not possible to say with five cases that SARS‐CoV‐2 can trigger anorexia nervosa; however, the connection between the disorder and the virus needs to be explored. In the end, it is important to keep in mind that people with history of eating disorder, or other mental disorders may be more susceptible to newly develop or exacerbate eating disorders after COVID‐19 infection especially when symptoms are gastrointestinal dominant.

## AUTHOR CONTRIBUTIONS

**Soode Tajik Esmaeeli:** Conceptualization; data curation; investigation; project administration; supervision; writing – review and editing. **Zahra Rahimi:** Conceptualization; investigation; methodology; project administration; software; validation; writing – original draft; writing – review and editing. **Fahimeh Saeed:** Conceptualization; data curation; investigation; project administration; validation. **Sheikh Shoib:** Conceptualization; investigation; methodology; validation; writing – review and editing.

## CONFLICT OF INTEREST STATEMENT

The authors declare that they have no conflict of interest.

## CONSENT

Written informed consent was obtained from patients/their parent/guardians to publish this report in accordance with the journal's patient consent policy.

## Data Availability

The data that supports the findings of this study are available in the supplementary material of this article.
